# Intermedia Agenda Setting amid the Pandemic: A Computational Analysis of China's Online News

**DOI:** 10.1155/2022/2471681

**Published:** 2022-04-16

**Authors:** Hanxiao Wang, Jian Shi

**Affiliations:** ^1^School of Journalism and Communication, Nanjing Normal University, Nanjing 210097, Jiangsu, China; ^2^S.I. Newhouse School of Public Communications, Syracuse University, Syracuse 13210, NY, USA

## Abstract

Based on Intermedia Agenda Setting (IAS), the current study examines how official media and semi-privatized commercial media on the Weibo platform covered the COVID-19 pandemic in China. Both supervised machine learning and time series analysis were employed to analyze 350,059 Weibo posts released by 3,883 news sources between December 2019 and April 2020. Our results indicated that, in this nonwestern state-regulated China media environment, official and semi-privatized commercial media had a significant reciprocal relationship in news coverage. Both of them focused on “treatment on patients,” “work resumption,” and “propaganda and mobilization.” Importantly, this paper sheds light on the value of the fine-grained level of agenda in IAS research. Using a fine-grained analysis, we separately investigated the effects of official and semi-privatized commercial media on predicting the pandemic prevalence, referring to the number of confirmed cases reported in real time. Implications and future directions were further discussed.

## 1. Introduction

On January 30, 2020, the World Health Organization (WHO) declared the global coronavirus (COVID-19) outbreak a pandemic. With over 455,675,890 confirmed COVID-19 cases and 6,038,904 deaths reported worldwide by March 12, 2022, the overall effect of the pandemic has had a huge impact on peoples' lives [1]. To fight the virus, much of the world has taken vigorous action to develop new vaccines. Many countries are pressing ahead, in a concerted response, with administering available vaccines in order to limit the spread of the virus. Although scientific experts and government officials have been furnishing the public with essential health guidelines, news media still play an important role in raising public awareness about healthy behaviors [2].

As such, many scholars are focusing their research on COVID-19 media coverage from multiple perspectives of news production and content [3, 4]. Utilizing a framing analysis, Jia and Lu [5] analyzed media coverage of COVID-19 in *The New York Times*, *The Wall Street Journal*, *the Washington Post*, and *Foreign Policy* and concluded that these four news outlets employed several main rhetorical techniques linking the virus and China, including “naming/shaming” (i.e., using labels suggesting that China should be associated with the virus), “blaming” (i.e., accusing the Chinese political system and food culture as causes of the pandemic outbreak), and “taming” (i.e., taking an approach to weaken China's international prestige). Primarily, Chinese scholars have examined different agendas among official media, semi-privatized commercial media, and other social media accounts (e.g., [6–8]). Extensive research analyzes the media coverage of hygiene security and infectious disease control using a traditional content-analysis method. However, this method can only provide a static analysis of health security and incidents of infectious disease [9, 10].

By contrast, this paper aims to provide a dynamic analysis of the COVID-19 pandemic from a nuanced look at the news constructed within the interactions of different media channels. This discussion emphasizes that public opinion does not simply comprise an amalgam of isolated or scattered opinions but also comprises the process of individual opinions interacting with one another. Specifically, we use Intermedia Agenda Setting (IAS) to examine whether media outlets set the agendas for one another while covering news [11].

In IAS studies, a crucial mediator for the selection of agenda items is granularity [12]. Granularity, commonly used to describe how detailed the data are, influenced the results and even the prediction of the trend of the COVID-19 pandemic. Considering the limited cost of acquiring data on media platforms, previous studies have extensively examined the relationship between social media data and pandemic prevalence [13, 14]. However, scholars sometimes draw different conclusions or even opposite conclusions toward similar topics. That might be because those studies introduce a coarse-grained approach [15]. In contrast, when it is verified that media have a predictive function for pandemic prevalence, using fine-grained categories of data helps to distinguish whether the prediction is caused by the media themselves or other factors. For example, in the cases of news coverage on “home quarantine” and “closure of public venues,” the media only covered messages released exactly by the government in regard to pandemic control.

Fine-grained analyses will critically influence the results of research that examine whether media outlets will influence each other in covering COVID-19 and whether the media have an impact on COVID-19 case trends [16, 17]. Still, using traditional content analysis to encode fine-grained analyses will necessitate higher requirements for both researchers and coders, significantly increasing the coding workload. Recently, scholars like Molina and Garip [18] advocate the application of machine learning in social-science research in order to achieve prediction through advanced techniques rather than traditional methods of description, interpretation, and statistical verification. With appropriate algorithms, the prediction will provide adequate information and evidence for the social decision-making process. It embraces the longitudinal prediction of inferring the future based on historical data and the horizontal prediction of inferring other data based on local data. Therefore, instead of employing traditional methods of coding, this paper combines traditional content analysis with the technique of machine learning. Specifically, we first conduct content analysis in order to obtain the fine-grained approach. Next, based on the coding results we obtained from content analysis, we use machine learning to predict the rest of our data.

Based on Intermedia Agenda Setting (IAS), the current study examines how official media and semi-privatized commercial media covered the COVID-19 pandemic. Additionally, we examine the extent to which media outlets follow one another in regard to the COVID-19 pandemic in the context of Chinese society. In order to analyze the influence of media on the COVID-19 pandemic, we collect both media data from the Weibo platform and domestic case data (i.e., the number of confirmed COVID-19 cases) between December 31, 2019, and April 16, 2020. Therefore, this particular discussion intended to highlight the scholarly imperative of investigating the interaction between various social media news sources, especially as that interaction pertains to the circulation of COVID-19 information via Weibo platforms. Importantly, this study seeks to address an important mediating factor, granularity, in IAS research and suggests using a fine-grained analysis to test the IAS in the nonwestern state-regulated society of China.

## 2. Literature Review

### 2.1. Media Environments in China

As several scholars have observed in the past two decades, Chinese media comprises a mix of the party-state and the commercial media systems (e.g., [19, 20]). China's online news environment, including social media accounts, has been classified into two types of media. One type consists of government-owned media and online forms of traditional media [21] such as Xinhua Agency and People's Daily, referred to as “official media” in the current study. Another type consists of privately owned media outlets [22] such as Sina, Sohu, and Caixin, referred to as “semi-privatized commercial media” in the current study.

As the “mouthpiece” of the Chinese Communist Party (CCP), the fundamental task of official media is to propagandize the principles and policies of the party and to ensure the execution of central tasks of the Communist party [23]. Official media are mainly composed of three types of media. Central-level media is the first of these types; it represents the central and local committees of the party, such as the People's Daily, Jiefang Daily, and Economic Daily. Professional and specialized media are a second type; examples include China Youth Daily and China Sports Daily. Provincial and municipal media represent a third category; morning, evening, and urban newspapers named by a province or a city fall under this category. To note, the provincial and municipal media are fundamentally different from semi-privatized commercial media. While provincial and municipal media are products of the marketization of China's newspaper industry, they maintain the original state-owned ownership, the same political position of the CCP, and previous editorial policies, which eliminate their dependence on governmental funding sources and the financial support of administrative institutions [24]. Therefore, in the current study, we categorized provincial and municipal media as one type of official media.

In China, semi-privatized commercial media emerged in the late 1990s. With the development of the Internet as an important news source for audiences, a number of commercial news websites, funded by nongovernmental capital, are emerging in the media market, resulting in the sharing of market resources with official media. One of the fundamental differences between official media and semi-privatized commercial media lies in the permission to deal with interviews. According to the *Regulations on the Administration of Internet News and Information Services* released by China's State Internet Information Office in May 2017, Chinese semi-privatized commercial media have not been granted the same rights as official media to cover news of the pandemic, nor have they been allowed to participate in any pandemic-related press releases discussing government-coordinated prevention and control. [21]. However, these media regulations were not fully implemented in reality.

In the Chinese media system, when facing an emergency like COVID-19, official media are given authority to investigate, and semi-privatized commercial media are under the regulation of the government. Under the influence of China's political system, official media have been playing a dominant role in the development of China's media industry [25]. However, semi-privatized commercial media do publish original content by offering additional information as an alternative to official propaganda (for details of semi-privatized commercial media in China, see [26]). For example, some semi-privatized commercial media like Sina covered stories that addressed the severity of the COVID-19 pandemic during the prepandemic period between January 1 and 20, 2020 [27]. As a result, both Chinese official media (e.g., People's Daily, Xinhua News Agency, and CCTV) and semi-privatized commercial media (e.g., Caixin and Sanlian Life Weekly) news articles on social media platforms have played a role in covering the pandemic. Thus, we posit the following two research questions:

- RQ1: What have been the agenda attributes of official media organizations on the Weibo platform during the COVID-19 pandemic?
- RQ2: What have been the agenda attributes of semi-privatized commercial media organizations on the Weibo platform during the COVID-19 pandemic?

### 2.2. Intermedia Agenda Setting (IAS) and Granularity

Based on pre-existing studies, agenda-setting effects are examined by the salience of media agenda and the salience of public agenda [28]. The salience of the public agenda can be measured by survey data, interviews, and even social-media data [29, 30]. Agenda-setting theory examines how media agendas set public agendas and acknowledges the strong relationship between the specific issues being emphasized by the media and the important issues in mass audiences' minds [11, 28]. In other words, the media can tell people what to think about through the selection of issues covered in the news. Intermedia agenda setting addresses the extent to which media outlets set the agendas for one another while covering news [11]. In the emerging media age, new technology has made exchanging information between news outlets convenient [31]. Media sources can and do monitor the reporting of their peers to avoid missing any important news events [32, 33]. This is especially the case for small- and medium-sized media with scarce resources to generate original news and highlight news values [34, 35]. For example, utilizing eighteen elite left-leaning and right-leaning political blogs and two newspapers (*The New York Times* and *The Washington Post*) with their eleven political newsroom blogs, Meraz [31] demonstrated that traditional media agendas failed to set political blog agendas during the period of July 20 to September 30, 2007, suggesting that political blogs were still able to influence the traditional media's agenda.

A crucial mediator for the selection of agenda items, which was defined as granularity, is usually ignored in IAS studies [12]. Granularity refers to “the grains, or components, of which something is composed or into which something can be divided, and is commonly used to describe the level of detail in data analysis” [12], p.28). Scholars further distinguish types of granularity as fine-grained and coarse-grained analyses [36]. A fine-grained analysis is defined as an analysis of specific components, whereas a coarse-grained analysis refers to the usage of larger components. To examine news agenda items, fine-grained and coarse-grained analyses indicate different levels of specificity of news coverage [12].

Although scholars have suggested that a coarse-grained analysis would underestimate the IAS effect, only a few studies have considered fine-grained-level IAS studies. That is because fine-grained-level IAS analysis usually requires more content analysis and complicates the subsequent statistical analysis [12]. Based on this argument, this study conducts a fine-grained analysis using content analysis and machine learning to code big data on Weibo and then to evaluate the accuracy and recall rate of the coding.

### 2.3. IAS in China's Media Environment

Although a majority of IAS studies have been researched in western countries, a few studies have also tested IAS effects in a state-regulated nonwestern media environment, such as the media environments of Chinese society (e.g., [37]). When comparing the official websites of traditional media (Renmin and Xinhua Websites) with semi-privatized commercial media websites (Sina and NetEase News) in the environment of China, Jiang and Deng [38] found that traditional media still had a strong impact on setting the agenda for semi-privatized commercial media in China. This impact was likely due to semi-privatized commercial media's lack of interview rights. Jiang and Deng [38] also proposed a two-level flow model in which the salience of topics transfers from the official mainstream news media to online media and then flows to the netizen.

Additionally, based on a Latent Dirichlet Allocation (LDA) model, Sun et al. [39] investigated 21,834 topics from Weibo (i.e., Twitter in the U.S.) media accounts. Their study summarizes a significant difference between official media and semi-privatized commercial media on the Weibo platform in the news agenda concerning the pandemic. Specifically, official media emphasizes national discourses and social mobilization from a macro level, while semi-privatized commercial media focuses more on microlevel changes, covering subjects relevant to an international community, focusing on, to a greater degree, people and events related to COVID-19. In Chinese society, only official media sources are authorized to collect information first-hand from government sources.

In contrast, Wang's [40] study found that in both crisis and noncrisis cases, Chinese official media failed to lead the discussion on the Weibo platform. Similarly, using an IAS analysis, Guo [37] found that China's official and commercial news websites, when it came to covering the *Two Sessions*, had a significant reciprocal relationship in news coverage, which suggested that official media did not necessarily set the agenda for semi-privatized commercial media in this highly controlled media environment. Thus, we propose a reciprocal relationship hypothesis:

- H1: While covering the COVID-19 pandemic, there will be a reciprocal agenda-setting relationship between the official news organizations and commercial news organizations.

### 2.4. Media Attention, Public Attention, and COVID-19 Prevalence

Media attention has been defined and measured by the prominence and amount of media coverage that an object receives [41, 42]. Several key factors can indicate the prominence of media coverage, including the length and placement of an article and the significance of the issue or actors mentioned within it (e.g., [43, 44]). Similar to other epidemics [45], the current COVID-19 pandemic has drawn widespread media attention. Media attention has been found to be associated with public perceptions and mass behaviors and even the number of confirmed pandemic cases [46, 47].

During the COVID-19 pandemic, another set of offline data has been available to evaluate the trend of the COVID-19 pandemic, which is the number of suspected and confirmed cases released by the National Health Commission of the PRC (2021). Tchuenche and Bauch [48] find that the coverage of the COVID-19 pandemic can have a subsequent influence on disease prevalence. Similarly, previous research has compared the data from search engines (e.g., Baidu index, Google index) and social media (Weibo, Twitter) with the data of confirmed cases to verify whether media coverage can impact COVID-19 prevalence. The current study utilizes data from confirmed COVID-19 cases in China, released by the Chinese government, and proposes the following hypotheses.

- H2: Official media agendas significantly predict confirmed COVID-19 cases.
- H3: Semi-privatized commercial media agendas significantly predict confirmed COVID-19 cases.

## 3. Methods

### 3.1. Data Collection

In order to better understand the IAS dynamics between China's official media and semi-privatized commercial media, two sets of data are used for the subsequent analyses—the data of media content from Weibo platforms and the data of domestic COVID-19 cases. The media data are collected from Zhiwei, a data collection company specializing in social-media data collection in China. The Zhiwei platform, widely used by scholars in various fields, collects data from Weibo, the WeChat public account, and all other news websites [22, 49]. Zhiwei officially classifies all news stories concerning the same topics under a single “news topic” category and provides a comprehensive news dataset on different topics.

The media data in this paper are collected from a news topic in Zhiwei, “The COVID-19 Pandemic in Wuhan and Other Places,” between December 31, 2019, and April 16, 2020. This period witnessed the most serious coronavirus outbreak in China, making these media texts extremely relevant. During this period, a total of 350,059 Weibo posts uploaded by 3,883 media sources (each media source is certified by Weibo Official) were collected. Based upon automatic labels collected from Zhiwei, 2,598 Weibo accounts of official media and 1,285 Weibo accounts of semi-privatized commercial media were detected. After this period of the pandemic outbreak, the Wuhan people were allowed to leave, and the domestic pandemic de-escalated with fewer confirmed cases. Data on confirmed coronavirus cases derive from real-time case numbers reported by national, provincial, and regional Health and Wellness Commissions from January 19 to April 16, 2020, in accordance with the period of collecting media data.

### 3.2. Manual Content Analysis

This study first conducted a conventional manual content analysis. Initially, a random sample of 10,000 Weibo posts was manually coded. Based on our work on previous studies of media coverage regarding the COVID-19 pandemic [39, 50], we initially adopted seven topic categories including “Pandemic Notifications,” “Treatment of Patients,” “Methods for Pandemic Prevention,” “Scientific Knowledge,” “Social Assistance,” “International Pandemic Situation,” and “Propaganda and Mobilization.” In order to be as comprehensive as possible, an additional news topic category, “Work Resumption,” was included for posts dealing with the restoration of the normal operation of the social economy, stabilization of employment, and protection of people's livelihoods.

Two graduate-student coders were trained to manually code for the type of media accounts (1 = official media, 0 = semi-privatized commercial media) and media attributes of the news coverage (1 = pandemic notification, 2 = treatment of patients, 3 = methods for pandemic prevention, 4 = scientific knowledge, 5 = social assistance, 6 = work assumption, 7 = international pandemic situation, and 8 = propaganda and mobilization). *Pandemic notification* coverage referred to stories that covered the number of new suspected cases, confirmed cases, and deaths reported by the national and provincial Health and Wellness Commission. *Treatment of patients* coverage referred to stories that covered where the infected patients were accepted, how they were cured, and whether they were rehabilitated. *Methods for pandemic prevention* coverage referred to stories that covered measures to control the spread of the pandemic. *Scientific knowledge* coverage referred to stories that covered symptoms and features of the virus, prevention and treatment options, and refutations on rumors or fake news. *Social assistance* coverage referred to stories that covered disclosure of information regarding donations and social demands. *Work resumption* coverage referred to stories that covered measures to restore normal operations of the society, especially to resume labor in and production by each industry and classes for school students. *International pandemic situation* coverage referred to stories that covered COVID-19 spreading trends, epidemic situations, and prevention and control strategies in foreign countries. *Propaganda and mobilization* coverage referred to touching stories about progressive individuals in fighting against the COVID-19 pandemic. A majority of Weibo posts (a maximum of 140 words in length) contain only one topic. If a Weibo post discussed two or more topics, we categorized the category as the one that contained the most. The intercoder reliability reached 0.885 and 0.764 Kappa coefficient tests, respectively.

### 3.3. Supervised Machine Learning

After accomplishing the manual coding on 10,000 random microblogs, these amounts of posts were used for building SVM models and were used to code the remaining 340,059 Weibo posts released by 3,883 media accounts. As the core essence of auto-coding, machine learning can automatically understand input data through mathematical models. Once the machine is fitted with a model with tunable parameters that can be adapted to the observed data, the process of *learning* begins. If patterns in the old data are observed by the program, it can predict and interpret new, relevant observations. The algorithm observes the output (*y*) of each input (*x*). Support vector machine is used to train and predict the relevant texts.

To evaluate the performance of the model, we divided the labeled news posts into “training set” (80%) and “testing set” (20%). Specifically, based on the training set, the SVM model was utilized to predict the news topic presence in the testing test. The performance of the models (i.e., topics) turned out to be acceptable. The classification performance was displayed in Table 1.

### 3.4. Time Series Analysis

The granger causality test, as a kind of time series analysis, is widely used in agenda-setting research [52]. To illustrate this approach, if a measure *y* could be better predicted from combining past values of *x* and *y* than from past values of *y* alone, we called the measure *x* the “Granger cause” of a measure *y*. This study produces a time series analysis to assess if IAS between official media organizations and semi-privatized commercial media organizations was present. We perform Granger causality models for eight topics between the two different media types. Based on four analytical criteria (Akaike, Hannan–Quinn, Schwarz, and the forecast prediction error), we determine a suitable time lag in the Granger causality test as 24 h [53]. Before the Granger causality test, we conducted the stationary time series and cointegration test to calculate the optimal order of time series. Then, the Granger causality test is applied to test the impact of two types of media on pandemic confirmed cases. To note, granger causality reflects the sequence of time instead of reflecting the real causality [54].

## 4. Results

### 4.1. Salient Topics in Official Media and Semi-Privatized Commercial Media

Table 2 illustrates the eight most salient topics in official media's and semi-privatized commercial media's coverage of the COVID-19 pandemic, identified by supervised machine learning. Overall, we found a considerable degree of similarity in covering the pandemic between the two types of media. “Work resumption,” “pandemic notifications,” and “methods for pandemic prevention” are the three most salient topics on both official and semi-privatized commercial media on Weibo platforms. By January 27, the number of Weibo posts on the topic of “work resumption” released by official media and semi-privatized commercial media rapidly increased to around 600 and 100, respectively, which were mainly focused on the supply of masks and food. By February 12, the official media and semi-privatized commercial media released over 1,000 and 200 posts daily related to the three topics, respectively, comprehensively reporting the measures taken in different areas to resume work. RQs 1 and 2 were both answered.

As mentioned above, when it comes to reporting domestic health issues and safety events in the past, official media focused more on risk control, whereas semi-privatized commercial media tend to cover more information related to social assistance. However, the current study showed that “social assistance” and “work resumption” were not the dominant topics for news on the COVID-19 pandemic. The correlations between official media's and semi-privatized commercial media's topics are 0.9, which means that the two media types are highly correlated with each other. In sum, the agendas between two media may be influenced by each other interactively. The information flow between the two media will be discussed in the following sections.

### 4.2. The Intermedia Agenda Setting (IAS) Analysis

The results of the Granger causality analysis indicated that only the topic of “social assistance” fails the test of stationarity and other topics all pass the test; therefore, we did not include this category in the subsequent analysis. As shown in Table 3, the Granger causality analysis provided information about the interaction between official media and semi-privatized commercial media. Overall, our results showed that official media and semi-privatized commercial media have interacted with each other on a variety of topics including “treatment on patients,” “work resumption,” and “propaganda and mobilization.” Official media do not necessarily set the agenda for semi-privatized commercial media on every single topic. In contrast, semi-privatized commercial media mainly dominate the agenda of official media on the topic of “methods for pandemic prevention.”

During the COVID-19 pandemic, the advantages of official media, which are authorized to obtain first-hand information exclusively from the government, were not fully demonstrated as expected. Even if official media took the leading role on some topics, the official media were simultaneously affected by semi-privatized commercial media on the same agendas in return. In sum, our study finds a statistically significant reciprocal relationship between official media and semi-privatized commercial media.

### 4.3. The Influence of Media on the Pandemic Prevalence

Hypothesis 2 tested whether the agendas of official media were more likely to predict confirmed COVID-19 cases.  Table 4 illustrates the Granger causality analysis results on the relationship between official media agendas and the number of confirmed COVID-19 cases. The official media agendas had an impact on the number of confirmed COVID-19 cases among five topics, namely, “pandemic notifications,” “treatment on patients,” “methods for pandemic prevention,” “scientific knowledge,” and “social assistance.” In the meantime, the results also showed that the official media agendas and the number of confirmed COVID-19 cases reported had reciprocal relationships in the topic categories of “pandemic notifications,” “treatment on patients,” and “social assistance.” However, the agendas of official media were more likely to predict the numbers of confirmed COVID-19 cases, especially in discussing the topic of “social assistance.” Given that more than half (5 out of 8) of the topics showed statistically significant results, H2 was partially supported.

Hypothesis 3 tested whether the agendas of semi-privatized commercial media tended to predict confirmed COVID-19 cases.  Table 5 illustrates the Granger causality analysis results on the relationship between semi-privatized commercial media agendas and the number of confirmed COVID-19 cases. Semi-privatized commercial media had an impact on the number of confirmed COVID-19 cases among six topics: “pandemic notifications,” “treatment of patients,” “methods for pandemic prevention,” “scientific knowledge,” “social assistance,” and “propaganda and mobilization.” The results also showed that the semi-privatized commercial media agendas and the number of confirmed COVID-19 cases had reciprocal relationships in the topic categories of “pandemic notifications,” “methods for pandemic prevention,” “scientific knowledge,” and “social assistance.” However, the semi-privatized commercial media agendas were more likely to predict the numbers of confirmed COVID-19 cases, especially in discussing the topics of “methods for pandemic prevention” and “social assistance.” Given that six (out of eight) topics showed statistically significant results, H3 was partially supported.

## 5. Discussion

During the COVID-19 pandemic, a series of mandatory regulations, such as home isolation and travel bans, were implemented in China. Social media has become one of the main channels for the public to obtain pandemic-associated information. This paper looks into the IAS effects on the COVID-19 outbreak in China. We collected news posted by official media and semi-privatized commercial media from the Weibo platform during the COVID-19 pandemic and classified the posts based on fine-grained approaches. Using a fine-grained analysis, we separately investigated the effects of official and semi-privatized commercial media on predicting the pandemic prevalence, referring to the number of confirmed cases reported in real time. This paper sheds light on the value of the fine-grained level of agenda in IAS research: our results found that, although a single topic can prominently affect media agendas, its impact may be underestimated when being mixed with other topics (i.e., coarse-fined approaches).

As mentioned above, official media are mainly represented by People's Daily, Xinhua News Agency, and CCTV, and commercial media are mainly represented by Caixin and Sanlian Life Weekly. The current study showed that official media and semi-privatized commercial media demonstrate a considerable degree of similarity in covering the COVID-19 pandemic on the Weibo platform. Previous studies found that official media are more likely to cover the topic of “propaganda and mobilization,” and semi-privatized commercial media tend to focus more on “international pandemic situations” [39, 55].

In contrast, results in this paper showed that the topics of “work resumption,” “pandemic notifications,” and “measures for pandemic prevention” are the three most salient topics for both official media and commercial media. News coverage collected from semi-privatized commercial media on the topic “propaganda and mobilization” ranks fourth, which is much more than that of the topic “international pandemic situations.” Additionally, both official media and semi-privatized commercial media covered news on topics of “work resumption” and “measures for pandemic prevention,” providing information following the demands of the public and considering the public safety and health as well as the stability of social and economic development. Furthermore, both types of media actively covered touching stories and narratives about progressive individuals fighting against COVID-19. For example, news stories like “a 50-Day Schedule of Academician Zhong Nanshan” and “a Doctor Who Had Postponed His Wedding to Fight Against the Pandemic” enhanced the public confidence in fighting against COVID-19.

China's online news environment changed rapidly in the past few years. Official media no longer have an edge in information acquisition. In the past, official media were privileged to acquire authoritative information from the government before releasing the information to commercial media [56]. In contrast, the current study found that official media and semi-privatized commercial media had a significant reciprocal relationship in covering the COVID-19 pandemic. In other words, topics on official and semi-privatized commercial media platforms highly interact with one another. Previous studies have shown similar results. For example, Wang [40] concluded that official media failed to lead the discussion on Weibo, whereas semi-privatized commercial media tended to be more influential than in the past in both crisis and noncrisis cases. The reasons can be twofold.

First, the CCP changed its way of notifying the public about public health and safety incidents. Currently, both the Chinese central government and local government have standardized the form and frequency of informing public health and safety events. The frequency of updating notifications is increased to more than twice a day [57]. For example, between Jan 27, 2020, and Jan 29, 2020, the Tianjin local government updated the pandemic notifications five times a day.

Second, the means of releasing news from the government have also been changed. In recent years, official media have their own social media accounts on various social network sites such as Weibo, WeChat, and Toutiao. Since February 2, 2020, Hubei officials held its press conference on a live video streaming platform. Live video streaming becomes the most popular way for China to report the COVID-19 pandemic situation and is beneficial for both commercial and official media [58]. In the past, official media was dominant because the information was transmitted from the government to the official media first and then published by official media. However, this advantage of official media has gradually decreased while the government changes the frequency and function of the news release.

Interestingly, based on the function of news coverage, the above eight categories of topics in this paper can be divided into two types. The first type includes topics that indirectly affect the public (indirect type), which means the information being covered, such as the “measures for pandemic prevention and control,” has already been released from the government and is well known by the society. The role of media in such a situation is to transmit the information. The second type includes the topics such as “scientific knowledge” and “propaganda and mobilization,” which directly affect the public in terms of their perceptions, attitudes, and even behaviors (direct type). Both indirect and direct information released by news sources has an impact on the pandemic prevalence. Compared with the indirect type, the direct type is more likely to affect the trend of the pandemic prevalence. For example, the direct type usually has an impact on the public's behaviors in regard to COVID-19 protective actions.

Overall, this study is innovative in researching IAS between official media and semi-privatized commercial media because it successfully avoids invalid results caused by the coarse-grained approach. In order to code a large volume of texts by fine-grained approach, this paper creatively combined the traditional content analysis method and machine learning technique.

We found a reciprocal relationship between the official media agendas and COVID-19 pandemic confirmed cases and a reciprocal relationship between the semi-privatized commercial media agendas and COVID-19 pandemic confirmed cases. The number of COVID-19 confirmed cases were found to predict media agendas of both official and semi-privatized commercial media. It might be because, during a pandemic, media attention was largely triggered by key events in reality [45]. Interestingly, both media agendas of official and semi-privatized commercial media also were found to predict the confirmed cases. This might be because media attention can not only determine what the public thinks but also contribute to public attention, which in turn influences mass behaviors [46]. Consistent with Tchuenche and Bauch [48], we also find a potential short-term beneficial effect of media coverage on the pandemic: the coverage of the COVID-19 pandemic can have a subsequent influence on disease prevalence. For example, people who were informed through news coverage that the number of cases had increased in a location avoided traveling to that place or reduced their contact rates with COVID-19 patients in the next few weeks.

Like other studies, this study is not without limitations. First, this paper only analyzed official media and semi-privatized media accounts on the Weibo platform but did not look into other news sources like “We Media,” opinion leaders, or the government account on Weibo, which have also participated in the discussion of relevant topics and played their roles in the information diffusion process. Similar to the difficulties mentioned by previous research [59], this paper cannot identify “We Media” accounts because of the dimensions of data and the large sample size.

Second, the confirmed COVID-19 cases cannot fully demonstrate the prevalence of the domestic pandemic. In the early stage of the COVID-19 outbreak, due to limited testing methods, statistics on confirmed cases released by the government were lower than the actual number in most countries around the world, including China. During the period of data collection in this paper, the COVID-19 case numbers might fluctuate abnormally in some days because those days were just the dates that missing cases were added. Thus, the results in this paper may be influenced by actual numbers. Third, based on the Granger causality analysis, this paper can only conclude that media have a potential impact on the trend of the pandemic. Future research should further examine whether the relationship between media and pandemic prevalence is positive or negative by using additional techniques [60].

## Figures and Tables

**Table 1:** Extended metrics for evaluating the performance of classifiers constructed by machine learning for the COVID-19 pandemic news coverage.

Topics	F1-score	Precision	Recall
Pandemic notifications	0.93	0.94	0.93
Treatment on patients	0.75	0.78	0.73
Methods for pandemic prevention	0.78	0.75	0.81
Scientific knowledge	0.81	0.79	0.83
Social assistance	0.69	0.80	0.60
Work resumption	0.85	0.82	0.87
International pandemic situation	0.90	0.89	0.92
Propaganda and mobilization	0.80	0.77	0.83

Note. We divided all the manually labeled Weibo posts into a training set and a testing set. Two tasks were taken for an SVM model. For the first task, we used the Weibo posts in the training set to build an SVM model. For the second task, we used the SVM model to predict the topic category in the testing set. To evaluate the performance of supervised machine learning, we compared the SVM-predicted labels and the manual labels by generating the precision and recall values, where precision=true positives/true positives+false positives and recall=true positives/true positives+false positives [51]. The prediction value measures how many of all the tweets predicted by the SVM model as this specific topic were indeed the same topic coded by manual coding. The recall value measures how many of all the tweets coded as this specific topic by manual coding were indeed predicted by the SVM model. Finally, an *F*-score is the weighted average of prediction and recall.

**Table 2:** Salient topics in official media and semiprivatized commercial media.

Order	Topics	Official media news amount	Order	Topics	Semiprivatized commercial media news amount
1	Work resumption	67740	1	Work resumption	11989
2	Pandemic notifications	61261	2	Pandemic notifications	8108
3	Methods for pandemic prevention	50860	3	Methods for pandemic prevention	7433
4	Propaganda and mobilization	40177	4	Propaganda and mobilization	6705
5	Scientific knowledge	28483	5	International pandemic situation	6381
6	International pandemic situation	20808	6	Scientific knowledge	5971
7	Treatment on patients	20586	7	Treatment on patients	2930
8	Social assistance	7990	8	Social assistance	2637

**Table 3:** Granger causality results for online news media of different types.

Topics	Semiprivatized commercial media ⟶ official media	Official media ⟶ semiprivatized commercial media
Pandemic notifications	1.10	3.22
Treatment on patients	3.63^∗^	3.09^∗^
Methods for pandemic prevention	3.55^∗∗^	1.36
Scientific knowledge	1.09	1.88
Social assistance	NA	NA
Work resumption	3.91^∗∗^	4.55^∗∗^
International pandemic situation	1.65	1.26
Propaganda and mobilization	4.52^∗∗^	3.56^∗∗^

*Note.* ^∗^ *p* < .05, ^∗∗^*p* < .01.

**Table 4:** Granger causality results for official media and confirmed cases.

Topics	Official media ⟶ confirmed cases	Confirmed cases⟶ official media
Pandemic notifications	3.97^∗∗^	2.87^∗∗^
Treatment on patients	2.87^∗∗^	6.22^∗∗^
Methods for pandemic prevention	3.07^∗∗^	1.76
Scientific knowledge	3.46^∗∗^	1.85
Social assistance	18.52^∗∗^	7.89^∗∗^
Work resumption	0.58	0.14
International pandemic situation	1.39	1.40
Propaganda and mobilization	1.70	1.95

*Note.* The Granger causality tests documented in the table are based on *F* distributions for significance. N/A indicates that at least one of the time series tested was not stationary ^∗^*p* < .05, ^∗∗^*p* < .01.

**Table 5:** Granger causality results for semiprivatized commercial media and confirmed cases.

Topics	Semiprivatized commercial media ⟶ confirmed cases	Confirmed cases ⟶ semiprivatized commercial media
Pandemic notifications	3.07^∗∗^	4.13^∗∗^
Treatment on patients	13.28^∗∗^	0.76
Methods for pandemic prevention	5.42^∗∗^	2.75^∗∗^
Scientific knowledge	5.30^∗∗^	5.31^∗∗^
Social assistance	9.25^∗∗^	5.87^∗∗^
Work resumption	NA	NA
International pandemic situation	1.45	1.33
Propaganda and mobilization	3.44^∗∗^	0.99

*Note.* The Granger causality tests documented in the table are based on *F* distributions for significance. N/A indicates that at least one of the time series tested was not stationary. ^∗^*p* < .05, ^∗∗^*p* < .01.

## Data Availability

The data that support the findings of this study are available from the corresponding author upon reasonable request.
